# Chemical agents transported by xylem mass flow propagate variation potentials

**DOI:** 10.1111/tpj.13624

**Published:** 2017-08-10

**Authors:** Matthew J. Evans, Richard J. Morris

**Affiliations:** ^1^ Computational and Systems Biology Crop Genetics John Innes Centre Colney Lane Norwich NR4 7UH UK

**Keywords:** variation potential, slow wave potential, systemic signalling, electrical signals, wounding, Arabidopsis, wheat, xylem flow, hydraulic waves

## Abstract

Long‐distance signalling is important for coordinating plant responses to the environment. Variation potentials (VPs) are a type of long‐distance electrical signal that are generated in plants in response to wounding or flaming. Unlike self‐propagating action potentials, VPs can be measured beyond regions of dead or chemically treated tissue that block signal generation, suggesting a different mode of propagation. Two alternative propagation mechanisms have been proposed: movement of a chemical agent and a pressure wave through the vasculature. Variants of these two signalling mechanisms have been suggested. Here, we use simple models of the underlying physical processes to evaluate and compare these predictions against independent data. Our models suggest that chemical diffusion and pressure waves are unlikely to capture existing data with parameters that are known from other sources. The previously discarded hypothesis of mass flow in the xylem transporting a chemical agent, however, is able to reproduce experimental propagation speeds for VPs. We therefore suggest that chemical agents transported by mass flow within the xylem are more likely than a pressure wave or chemical diffusion as a VP propagation mechanism. Understanding this mode of long‐distance signalling within plants is important for unravelling how plants coordinate physiological responses via cell‐to‐cell communication.

## Significance Statement

Variation potentials are a mode of electrical signalling that allow plants to communicate over long distances to coordinate responses to the environment. Here we use simple physical models to evaluate existing propagation hypotheses for variation potentials, raising questions about the pressure wave and turbulent chemical diffusion hypotheses and lending support to the model of mass flow within the xylem transporting a chemical agent.

## Introduction

Plants communicate from cell to cell and between distant tissues to coordinate activities in response to their environment. Long‐distance communication within plants involves a number of different signal propagation mechanisms and diverse signalling agents, including electrical (Fromm and Lautner, 2007; Vodeneev *et al*., 2016), hydraulic and chemical signals (Malone, 1992), depending on the perceived stress (Gilroy *et al*., 2014; Shabala *et al*., 2015). Wounding stresses, for example, trigger electrical signals (Mousavi *et al*., 2013; van Bel *et al*., 2014; Salvador‐Recatalà *et al*., 2014), waves of reactive oxygen species (ROS; Miller *et al*., 2009; Romeis and Herde, 2014), calcium transients (Kiep *et al*., 2015), and changes in vascular pressure and flow (Malone, 1996; Stahlberg and Cosgrove, 1997b; Christmann *et al*., 2013). We can divide the underlying signalling mechanisms into two groups: those that require living cells to propagate, typically because the signal is regenerated within each cell; and those that do not. The former includes signals such as a calcium wave (Choi *et al*., 2014) or a ROS wave (Miller *et al*., 2009) that can be locally blocked by chemical inhibitor treatments, the latter includes ‘hydraulic waves’ or diffusing chemical signals that can propagate through the metabolically inactive cells of the xylem.

Action potentials (APs) occur in response to non‐invasive stresses (van Bel *et al*., 2014) and utilise a self‐propagating mechanism: flux of ions through channels generates the AP in a cell, thereby also changing the membrane voltage of neighbouring cells and triggering the opening of voltage‐sensitive channels that leads to the generation of the AP in those adjacent cells (Sukhov and Vodeneev, 2009; Sukhov *et al*., 2011; van Bel *et al*., 2014). This process is analogous to signals in animal nerve cells and requires cells to be metabolically active to ensure propagation (Mancuso, 1999).

Variation potentials (VPs), also known as slow wave potentials (SWP), in contrast to APs, have the capacity to traverse regions of dead or poisoned tissue (Stahlberg *et al*., 2006). VPs are unique to plants and occur in response to wounding or flaming (Stahlberg *et al*., 2006). The initiation of VPs requires ionic fluxes (Vodeneev *et al*., 2011; Katicheva *et al*., 2014) and a decrease in H^+^ ATPase activity (Julien and Frachisse, 1992), but how they propagate is not understood.

The propagation mechanism underlying VPs must be capable of passively passing through regions of dead tissue (Stahlberg *et al*., 2006), thus excluding self‐propagating ionic species that form APs, ROS or calcium waves (Miller *et al*., 2009; van Bel *et al*., 2014; Choi *et al*., 2014; Evans *et al*., 2016) as the underlying mechanism. Preventing generation of the electrical signal in one cell does not prevent generation of the electrical signal in later cells. The measured VP, therefore, reflects the generation of successive depolarisations of each cell in sequence, which appear as an electrical propagation, but are in effect electrically uncoupled (Stahlberg and Cosgrove, 1997b; Vodeneev *et al*., 2012, 2015; Sukhov *et al*., 2013; van Bel *et al*., 2014). So, whilst VPs are electrical signals, their propagation mechanism is distinct from APs. VPs are thought to be local responses to another propagating signal, either a hydraulic wave (Malone, 1992; Stahlberg and Cosgrove, 1997b; Farmer *et al*., 2014) or a diffusing chemical signal (Ricca, 1916; Malone, 1996; Vodeneev *et al*., 2012) or both (Malone, 1996; Vodeneev *et al*., 2015).

It has been widely suggested that passage of hydraulic waves triggers VPs (Malone, 1992; Stahlberg and Cosgrove, 1997b; van Bel *et al*., 2014), plausibly through activation of mechano‐sensitive ion channels (Christmann *et al*., 2013). An alternative hypothesis, originally proposed by Ulrich Ricca a century ago (1916), is that a chemical substance, released into the vasculature upon wounding, triggers electrical signals in nearby cells (Malone, 1996; Vodeneev *et al*., 2012). Such a signal propagates by the chemical substance diffusing through the vasculature (Vodeneev *et al*., 2012, 2015). Neither of these mechanisms requires the surrounding cells to play an active role in their propagation, and so can propagate through dead tissues. Both proposed mechanisms give rise to testable predictions in terms of the behaviour of wave propagation and associated material properties.

Neither of these models, however, can directly explain the speed of apparent VP propagation. VPs typically propagate at speeds on the order of 1 mm s^−1^ (Choi *et al*., 2016), but the diffusion of chemical species is typically two orders of magnitude slower (Mastro *et al*., 1984), while an hydraulic wave would travel at about four–five orders of magnitude faster (Malone, 1993, 1996; Christmann *et al*., 2013). To account for the differences between hydraulic and electrical signal velocities, Stahlberg and Cosgrove (1997a,b) propose a mechanism that connects axial hydraulic waves to radial pressure and surface electrical signals. Stahlberg and Cosgrove (1997a,b) observed that pressure‐induced changes in epidermis electrical potential lag behind the hydraulic wave. This delay depended on the size of the applied pressure change, and on the position along the stem at which the electrical signal was measured. They proposed that the passage of the axial pressure wave triggered radially propagating signals, possibly in the form of pressure changes (Stahlberg and Cosgrove, 1997b), which activate ionic fluxes and therefore electrical signals in the epidermis. Malone (1996) examined how flow within the vasculature, directed away from the site of wounding, could be responsible for propagating a chemical signal at speeds in excess of those achievable by diffusion alone. Another suggestion for accounting for the discrepancy of speeds is that xylem turbulence could effectively increase the diffusion constant (Sukhov *et al*., 2013). In this paper, we examine these hypotheses through the use of simple mathematical models based on the underlying physical processes. The aim of these simple models is to provide an estimate for the likely order of magnitude of predictions from different hypotheses. We focus on the mechanism of the signal that triggers VP. Whilst pressure waves and chemical diffusion are likely to occur within the vasculature, we find that the pressure wave hypothesis (Stahlberg and Cosgrove, 1997b) and chemical diffusion (Sukhov *et al*., 2013) are unlikely to fit with known material properties of cells as a mechanism for VP propagation, whereas transport of a chemical agent by mass flow in the xylem (Malone *et al*., 1994) is able to provide much better agreement with experimental data.

## Results

### The hydraulic VP propagation model predicts that the velocity of electrical signals varies with distance from the xylem

**Figure 1:**
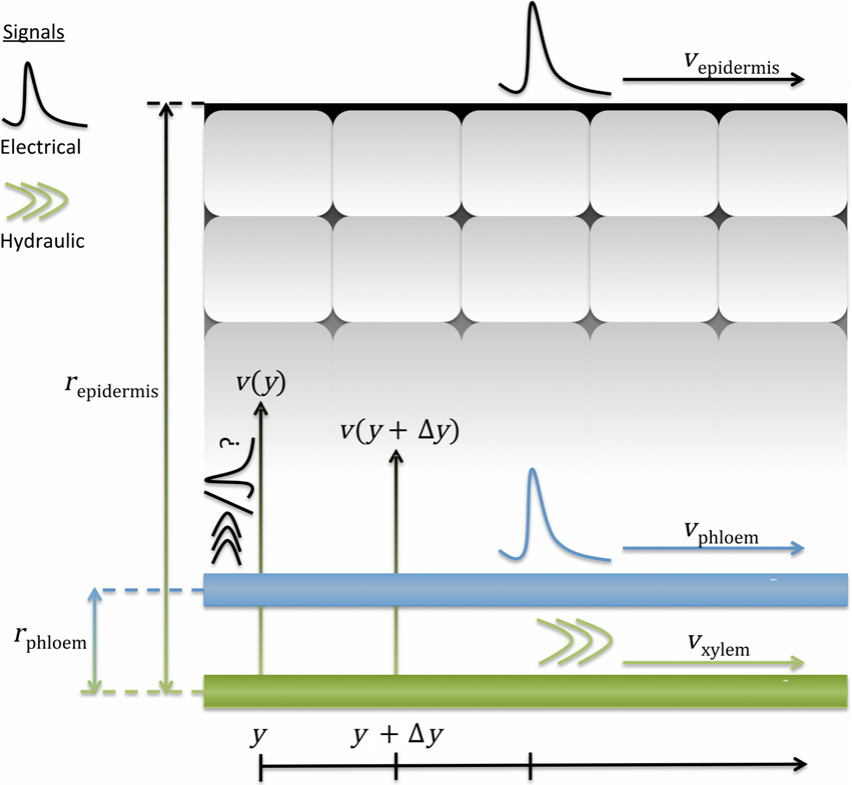
Model of electrical signal propagation driven by ‘hydraulic waves’. Propagation of an axial pressure wave through the xylem (with velocity *v*_xylem_) has been proposed to trigger electrical signalling at the plant epidermis and within the phloem. These electrical signals appear to travel at velocities of *v*_epidermis_ and *v*_phloem_, respectively. Differences in these velocities will occur if the signal propagating radially from the xylem varies with position, *v*(*y*) *≠ v*(*y + *Δ*y*), see main text for details. The phloem and epidermis are at a distance *r*_phloem_ and *r*_epidermis_ from the xylem, respectively.

**Figure 2:**
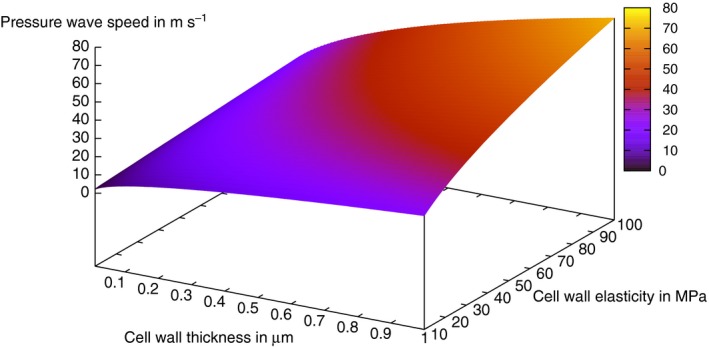
Predicted pressure waves speeds in the xylem. Predictions for pressure wave propagation speeds in the xylem as a function of cell wall thickness and cell wall elasticity, following the Moens–Korteweg equation. For the diameter of the xylem we used 50 μm and chose 1000 kg m^−3^ for the xylem fluid density. This plot shows that whilst propagation speeds can drop to about 1 m s^−1^, this requires very thin and highly elastic cell walls. Given estimates of cell wall thickness and elasticity from other studies, the pressure propagation speed is likely to be well above 10 m s^−1^.

Stahlberg and Cosgrove (1997a,b) suggest that axial hydraulic waves within the xylem might give rise to electrical signals at the epidermis and within the phloem by means of a radially propagating pressure change emanating from xylem vessels. This signalling process is illustrated in Figure 1. Approximating the xylem as a fluid within a cylindrical, elastic tube allows us to estimate the pressure wave propagation speed (Experimental procedures), following the Moens–Korteweg equation (Newman and Greenwald, 1978). This velocity is shown in Figure 2 for typical xylem parameters (McCulloh *et al*., 2003; Brodersen *et al*., 2011), and over ranges of cell wall thickness and its elasticity. For a cell wall thickness of 10 μm (Brodersen *et al*., 2011) and elasticity of 100 MPa, we would expect pressure waves to propagate at velocities close to 100 m s^−1^. Malone (1993) and Stahlberg and Cosgrove (1997b) point out that hydraulic waves approach the speed of sound and travel several orders of magnitude faster than VPs, which travel at about 1 mm s^−1^. To account for this difference in speed of propagation between pressure waves and VPs, Stahlberg and Cosgrove (1997b) suggests that a lag time between pressure wave propagation and surface membrane depolarisation might be induced by a radial pressure wave in the apoplast. A main argument of the hydraulic wave explanation of VPs is, thus, that an axial pressure wave through the vasculature, travelling with velocity *v*_xylem_, gives rise to a radial wave, which travels at velocities *v*(*y*) and triggers electrical signals with some lag time. This radial velocity might depend on the position, *y*, along the plant axis (Figure 1). If the behaviour of the radially propagating disturbance did not depend on *y*, then the velocity would be the same everywhere, *v*(*y* + Δ*y*) = *v*(*y*), and the surface electrical signal would appear to travel at the same speed as the axial hydraulic wave. Stahlberg and Cosgrove (1997a,b) proposed that the decreasing amplitude of the hydraulic wave might trigger slower radial disturbances. In this case, radial disturbances initiated further from the wounding site would propagate with a slower velocity, *v*(*y* + Δ*y*) < *v*(*y*), and delay times would increase with distance, *y*. When this radial wave reaches the surface, it causes the generation of the measured electrical signal. Assuming the radial velocity does not vary over these short distances, the delay time, *t* ^d^, between passage of the axial hydraulic wave and possible detection of an electrical signal at a radial distance, *r*, from the xylem is *t* ^d^(*r*,* y*) = *r*/*v*(*y*).

In this model in which the driving axial pressure wave propagates through the vasculature and activates a radial wave towards the epidermis, the expected delay time depends on *r*, the radial position at which the electrical signal is measured. Electrical signals in response to wounding have been measured on both the epidermis (Mousavi *et al*., 2013) and in the phloem (Salvador‐Recatalà *et al*., 2014). Because the delay time scales with distance from the xylem, the delay time that would be measured in the phloem would be less than the delay time measured at the epidermis

(1) $$t_{\mathrm{phloem}}^{d}=\frac{r_{\mathrm{phloem}}}{r_{\mathrm{epidermis}}}t_{\mathrm{epidermis},}^{d}$$

assuming the radial velocity is constant for a given point on the axis, *y*. If there is a decay in the velocity of the radial pressure disturbance during propagation, the difference in delay times would be further exaggerated. From this we would expect a faster signal to be measured in the phloem compared with the surface. Based on the model in Figure 1, the velocities would be expected to scale according to the relative radial distances

(2) $$v_{\mathrm{phloem}}=\frac{r_{\mathrm{epidermis}}}{r_{\mathrm{phloem}}}v_{\mathrm{epidermis}.}$$

Within the Arabidopsis stem, the value of *r*_epidermis_/*r*_phloem_ is in the region of 5 to 10 (Ibanes *et al*., 2009), while in the rosette leaf petiole, *r*_epidermis_ is at least 10 times *r*_phloem_ (Tian *et al*., 2010). The hydraulic wave hypothesis would therefore predict velocities for electrical signals in the phloem that are 5 to 10 times the speed of VPs measured at the epidermis. Stahlberg and Cosgrove (1997b) suggested that it is changes in cell turgor that trigger the VP. These changes take longer to occur the further from the xylem they are measured (Westgate and Steudle, 1985). There is a decay in radial propagation. However, this decay would increase the difference between phloem and xylem signal speeds.

Mousavi *et al*. (2013) measured surface electrical signals that appeared to propagate with at speeds of 0.8–1.5 mm s^−1^ in response to leaf excision at the tip, whereas Salvador‐Recatalà *et al*. (2014) measured the velocity of electrical signals in the phloem in response to chewing insects (1 mm s^−1^) and cutting (2.5 mm s^−1^). In order for phloem and epidermal electrical signals to have approximately the same apparent velocity, the radial signal velocity must be the same at all points along the plant axis, *v*(*y* + Δ*y*) = *v*(*y*). However, this would imply that both phloem and surface electrical responses propagate at the same speed as the underlying xylem signal. Thus, experimental measurement of VP speeds in Arabidopsis does not support the predicted differences. We conclude that the proposed hydraulic axial wave coupled with radial pressure disturbances that result in electrical signals does not solve the problem of hydraulic waves being orders of magnitude faster than observed VP propagation speeds.

### Diffusion is unlikely to account for known VP propagation speeds

We have discussed the consequences of the hydraulic model for VP propagation. An alternative way in which VPs might be propagated is through the movement of a chemical signal (Ricca, 1916; Malone *et al*., 1994; Malone, 1996; Vodeneev *et al*., 2012). The migration of molecules could trigger electrical reactions in cells adjacent to the vasculature, which could propagate radially to the epidermis in the same way that an AP propagates (van Bel *et al*., 2014), or the chemicals themselves could diffuse from the xylem through the apoplastic space, initiating electrical signalling in adjacent cells. Vodeneev *et al*. (2012) suggested a model in which a chemical species diffuses through the vasculature that then trigger electrical signals. Diffusion constants of small molecules in cellular environments are in the order of 10^−6^ cm^2^ s^−1^ (Mastro *et al*., 1984). The time, *t*, for a molecule with a diffusion constant of *D* to diffuse a distance *x* can be estimated from

(3) $$t=x^{2}/2D.$$

The velocity *v = x*/*t = *2*D*/*x* thus depends on the distance. For the movement over 10 mm, we would expect a diffusion time of *t *= 50 000 s and an average speed of *v* = 0.0002 mm s^−1^ compared with the experimental value of about 1 mm s^−1^. In order to obtain good fit of their model to VP propagation data, Vodeneev *et al*. (2012) determined a theoretical diffusion constant of 4.5 × 10^−2^ cm^2^ s^−1^, orders of magnitude greater than would be expected experimentally (Mastro *et al*., 1984). Their explanation for how this high diffusion constant may arise is that xylem flow may be turbulent (Vodeneev *et al*., 2012, 2015). Turbulent flow can be orders of magnitude faster than diffusion, resulting in speeds in good agreement to those of VPs (Vodeneev *et al*., 2015). The nature of fluid flow is characterised by Reynolds' number (Reynolds, 1883), which is a dimensionless number that quantifies the relative importance of inertia to viscous forces. For flow within a smooth tube, a Reynolds number greater than 4000 is characteristic of turbulent flow and numbers smaller than 2000 are typical for laminar flow. The Reynolds number for xylem flow (Ellerby and Ennos, 1998) can be estimated from the equation for flow in a smooth pipe

(4) Re=ρvd/μ

where ρ is the fluid density, *v* the velocity, *d* the xylem diameter and μ the dynamic viscosity. Using ⍴ = 1000 kg m^−3^, *v *= 1 mm s^−1^, *d *= 50 μm and μ* *= 10^−3^ Pa s^−1^, the approximate Reynolds number of flow in the xylem is 5 × 10^−2^, consistent with previous reports (Rand, 1983; Roth, 1996; Ellerby and Ennos, 1998). For a Reynolds number this low, the effect of pipe roughness is negligible (Moody, 1944); however, varying xylem profiles can modify the behaviour. The profile of the xylem is known to vary in diameter, contain ring thickenings and deviate from a circular cross‐section. Non‐circular cross‐sections require corrections to the flow rate from the Hagen–Poiseuille equation (Lewis and Boose, 1995); however, for nearly cylindrical tubes (Lee *et al*., 2013) the errors are small. In Roth (1996), the varying profile of the xylem caused by ring thickenings was taken into account to compute the flow field. Interestingly, slow circulation zones emerged between these thickenings that reduce the overall flow through the xylem. As is the case for cytoplasmic streaming, accounting for realistic geometries can produce seemingly complex yet laminar flows (Pickard, 2003; Goldstein and van de Meent, 2015). We conclude that turbulent xylem flow and a VP propagation mechanism based on chemical movement driven by turbulent diffusion is unlikely.

### Chemical agents transported by vascular flow can recapitulate VP propagation speeds

The transport of chemicals has been identified as having a role in systemic acquired resistance (Shah *et al*., 2014), and the movement of small molecules is consistent with the ability of VPs to pass across regions of dead tissue (Stahlberg *et al*., 2006). Although diffusion is unlikely to be sufficient as a mechanism for chemical transport for VP propagation, mass flow has long been suggested as a possible means to move chemical agents through the vasculature (Malone *et al*., 1994). Xylem flow could carry chemical messengers through the plant and trigger electrical signals in nearby cells. The concentration of these chemical agents could be reduced through binding events, which can be accounted for with an effective decay rate. For a flow rate, *v*_xylem_, along the *y*‐axis (Figure 1), diffusion rate, *D*, and decay rate, *K*, the flux of a chemical species obeys the advection–diffusion–decay equation (Carslaw and Jaeger, 1959)

(5) $$\frac{\partial c}{\partial t}+v_{\mathrm{xylem}}\frac{\partial c}{\partial y}=D\frac{\partial^{2}c}{\partial y^{2}}-Kc,$$

where *c* is the concentration of the species. This equation (5) can only be solved analytically in very few cases, but for an instantaneous release of a substance at time *t* = 0, the prototypical solution (Carslaw and Jaeger, 1959) is given by

(6) $$c\left(y,t\right)=\frac{M}{\sqrt{4\pi Dt}}e^{-\left(\frac{{\left[y-v_{\mathrm{xylem}}t\right]}^{2}}{4Dt}+Kt\right)},$$

where *M* is the total amount of substance released. Triggering generation of a VP within a cell in this model would require some threshold concentration of the chemical, which we denote *c*_th_, representing the quantity of the species required to activate sensitive channels. The threshold *c*_th_ is equivalent to the equilibrium dissociation constant for the chemical agent to the receptor that initiates the electrical response (Sukhov *et al*., 2013). Setting *c*(*y*,* t*)* = c*_th_ and solving for *y* gives the distance from the wounding site, *y*_th_, at which the chemical has just reached this concentration

(7) $$y_{\mathrm{th}}\left(t\right)=v_{\mathrm{xylem}}t+\sqrt{4Dt\left(\text{log}\frac{M}{c_{\mathrm{th}}\sqrt{4\pi Dt}}-Kt\right)}$$

**Figure 3:**
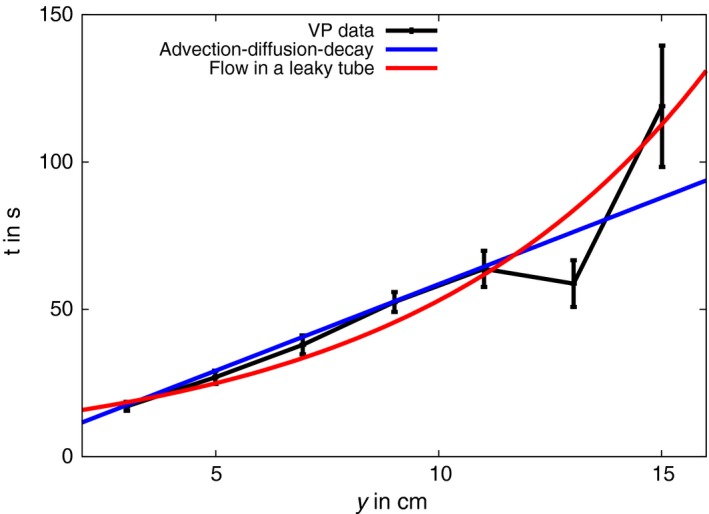
A model of xylem mass flow fits variation potential (VP) propagation data from wheat. Experimental measurement of surface electrical changes (black line) in response to burning leaf tips of wheat, reproduced from Vodeneev *et al*. (2012). The distance along the longitudinal axis of the leaf (*y*, as in Figure 1) is plotted against the time after burning, *t*. A model of chemical diffusion in the presence of background xylem transport (blue line) produces a good fit to these data with parameters that are consistent with reported values (see main text for details). A model for flow within a leaky tube is shown (red), which accounts for slower speeds at increasing distances. This curve was obtained by fitting equation (8) for flow at the centre of the xylem (*r* = 0).

For a small molecule, a typical diffusion rate is *D *= 10^−6^ cm^2^ s^−1^ (Mastro *et al*., 1984). Using this we can fit to the available data from Vodeneev *et al*. (2012) with a background flow velocity of 1.7 mm s^−1^ (Figure 3). The failure of the model to fit the final points of the data is likely due to our approximation using a constant flow velocity, which is an oversimplification as the flow away from the wound site will decrease as the fluid from the wound site is drawn into the xylem (Malone, 1996). In the present case, advection dominates over diffusion and the first term in equation (7) gives rise to near linear behaviour (Figure 3, black curve), which cannot be corrected for by the decay term *K*. To explore further the observation that VP speeds decrease with distance (Stankovic *et al*., 1997; Stanković *et al*., 1998; Mancuso, 1999; Vodeneev *et al*., 2011, 2012), we used a model for the velocity in a leaky pipe (Gorji *et al*., 2011)

(8) $$v_{\mathrm{xylem}}\left(y\right)=2v_{0}e^{\left(-2\beta \frac{y}{d}\right)}\left(1-{\left(\frac{2r}{d}\right)}^{2}\right)$$

in which *v*_0_ is the average velocity at the inlet, β is a dimensionless parameter (a function of the dynamic viscosity, the permeability of the pipe and the pipe radius), *d* the xylem diameter, and *r* a radial point from the central axis of the pipe. A leaky pipe model is motivated by the xylem being able to exchange water with the phloem and water being able to evaporate from the xylem. Although simple, this model can be fit to the data (Figure 3, red curve) and captures the decrease in flow velocity with increasing distance. The inferred initial velocity from this model at the centre of the pipe (*r* = 0) was 3.8 mm s^−1^. Integrating equation (8) with the inferred parameters over the range of the data resulted in an average velocity of 1.66 mm s^−1^. Xylem flow rates will depend on the pressure differential and can therefore be expected to show significant variation. The xylem flow rate in wheat has been measured as 0.8 mm s^−1^ (Passioura, 1988), and in *Ricinus communis* at 0.4 mm s^−1^ (Peuke *et al*., 2001). Hydrostatic pressure differences upon wounding (Malone and Stanković, 1991) may induce higher mass flow velocities and pressure or flame induced waves tend to be faster (Stankovic *et al*., 1997), but the reported numbers for VP (Malone and Stanković, 1991; Malone, 1992, 1993, 1996; Malone *et al*., 1994; Mancuso, 1999; Vodeneev *et al*., 2011, 2012, 2015, 2016; Choi *et al*., 2016) are of a similar order of magnitude to the model. We thus conclude that speeds of xylem mass flow are consistent with experimental data on VPs.

## Discussion

The ability for VPs to pass through dead and poisoned tissue (Stahlberg *et al*., 2006) eliminates self‐propagating mechanisms such as calcium (Choi *et al*., 2014) or ROS (Miller *et al*., 2009) waves, which require generation of the signal in previous cells in order to be generated in the current and future cells. These species are likely involved in the generation of the VP depolarisation within individual cells (Katicheva *et al*., 2014), but this is likely a passive response to the passage of another signal. The identity of this signal could be hydraulic or chemical.

The hydraulic model of VP generation requires a radially propagating signal whose velocity decays with distance from the wound site. However, this would lead to significant differences in the velocity of epidermis and phloem VPs, which are not observed (Mousavi *et al*., 2013; Salvador‐Recatalà *et al*., 2014). The proposed mechanistic basis for a propagating radial disturbance requires a membrane potential change with turgor, which is not supported by experiment (Westgate and Steudle, 1985; Lew, 1996; Kim *et al*., 2010). Vodeneev *et al*. (2012) were able to put an upper limit on the speed of the axial signal by leaf sectioning post‐wounding. Sectioning the leaf 1 s after wounding prevented transmission of the VP, and suggests the axial signal cannot travel faster than 3 cm s^−1^, much less than the speed of a pressure wave (Christmann *et al*., 2013). On the other hand, a model of chemical signals transported by mass flow in the xylem approximately fits VP propagation data with the correct order of magnitude, 1.7 mm s^−1^ (model) compared with values frequently in the range of 0.4–2 mm s^−1^ (experimental; Sukhov *et al*., 2013). The precise values will depend on the pressure differences that drive mass flow and xylem geometry. Wounding cells makes large volumes of water and solute available to the xylem under atmospheric pressure. Given that the xylem is under tension, this can drive flow away from the wounding site (Malone, 1996). The initial flow velocity under these circumstances may well be higher than speeds measured in unwounded plants. Indeed, Vodeneev *et al*. (2012) demonstrated through experiments with radioactive tracers that translocation speeds after wounding were enhanced relative to non‐wounding.

Variation potentials can be propagated through a solution connecting two parts of a severed stem (Ricca, 1916) suggestive of chemical messaging. The requirement for plasmodesmata in systemic electrical signalling (Bricchi *et al*., 2013) further supports a chemical means of propagation as pressure disturbances can be transduced through mechanical stress of the cell wall. Chemicals indicating damage to the plant are known to trigger Ca^2+^ transients during pathogen attack (Ferrari *et al*., 2013), as well as suggested to underlie mechanical responses in *Mimosa pudica* (Ricca, 1916; Volkov *et al*., 2010), while a large number of stress‐induced xylem proteins was recently identified in cotton (Zhang *et al*., 2015). Molecules suggested to be involved in wound signalling include oligosaccharides or plant hormones such as systemin (Hlavackova *et al*., 2006; Ferrari *et al*., 2013). Stahlberg and Cosgrove (1997b) rejected the chemical agent hypothesis as their experiments using only pressure steps could recapitulate VPs without wounding and therefore excluded a wounding substance being present. However, a hormone such as jasmonate may either already be present or could be quickly synthesized (Hlavackova and Nauš, 2007; Mousavi *et al*., 2013; Farmer *et al*., 2014), and may not require wounding *per se*. During wounding, ions and chemicals stored within the vacuole could be released into the surrounding tissue and the vasculature, when the cell is damaged (Malone, 1996).

If VP propagation requires xylem flow rather than propagating pressure changes, as we have proposed, then we would expect xylem tension to be an important requirement for VP propagation. Wounding experiments performed under conditions of varying xylem tension, for example at night or under drought conditions, would be expected to show different rates of VP propagation. It has been shown that the release of xylem tension is important for long‐distance signalling and that positive pressure steps are required for VPs (Stahlberg and Cosgrove, 1995, 1997a). Malone (1996) demonstrated an absence of VPs under very low xylem tension conditions. Xylem tension would not be expected to affect propagation of a pressure wave unless it led to a substantial change in the material properties.

Bulk flow within the xylem as a transport mechanism for a chemical agent was rejected based on the observation of basipetal VP transmission (Vodeneev *et al*., 2015). However, if the xylem is under tension we might expect flow away from wherever the wounding site occurs. Also, recent high‐resolution computed tomography experiments have demonstrated that between 4 and 27% of xylem vessel segments in grapevine exhibited reverse flow compared with the bulk flow under normal transpiration conditions (Lee *et al*., 2013). Whilst reverse flow depends mainly on the number of vessel relays (which enhances vessel resistance), vessel endings and xylem connectivity, favourable situations for reverse flows are likely to occur for heterogeneous, well‐connected networks and can readily be observed (Lee *et al*., 2013). The connection between phloem and xylem and the likely free flow of water between them (Hall and Minchin, 2013) may offer a means of maintaining flows and refilling embolized conduits (Lee and Kim, 2008; Zwieniecki and Holbrook, 2009; Nardini *et al*., 2011).

In this report, we examined the physical implications of the hydraulic wave hypothesis (Farmer *et al*., 2014; Katicheva *et al*., 2014; Shabala *et al*., 2015). We point out that the term hydraulic wave is somewhat ambiguous and has been seemingly used to describe a change in turgor pressure moving through tissue (largely dependent on ion channel activity), xylem mass flow (largely dependent on pressure differences) and a pressure wave (largely a function of material properties), within the apoplast, the vasculature and cells.

The presented models are simple and make a number of approximations relating to the geometry and material properties of the surroundings of the medium in which the wave propagates. Such parameters are known to influence the propagation speed of pressure waves and mass flows, so our calculations should be viewed as estimates of the order of magnitude. We have used available models wherever possible and made simplifying approximations in order to obtain analytical solutions. Nevertheless, given these estimates and experimental data, we can conclude that pressure waves are unlikely to be directly responsible for the propagation of VPs. Likewise, we find that diffusion or turbulent diffusion of a chemical are not likely propagation mechanisms. In agreement with previous suggestions by others, we find that the models and available data support the hypothesis of a propagation mechanism for VPs that transmits a chemical signal by mass flow in the xylem. However, many questions remain unanswered, and further experimental and theoretical work is required to validate and explore in more detail the models investigated here.

## Experimental Procedures

### Pressure propagation in the xylem

We approximated the xylem as a fluid column of diameter *d* with an elastic wall of thickness *e* and an incremental Young's modulus *E*. The Moens–Korteweg equation,

(9) $$v={\left(Ee/d\rho \right)}^{1/2},$$

can be used to estimate the speed, *v*, of a pressure disturbance. Xylem diameters are typically in the range of 25–75 μm (Davis *et al*., 1999), but can vary as much as 18–139 μm (Brodersen *et al*., 2011), and the fluid density is close to that of water, ρ = 1000 kg m^−3^. For the cell wall thickness and its elasticity we explored a range of values.

### Curve fitting

Measurements of VP propagation in wheat (Figure 3) were fitted using a multi‐dimensional non‐linear optimisation function (Marquardt–Levenberg algorithm) from the GNU Science Library (Galassi *et al*., 2015).

## Conflict of Interest

The authors declare no conflict of interest.
